# Motor neuron disease risk and magnetic field exposures

**DOI:** 10.1093/occmed/kqab180

**Published:** 2021-12-23

**Authors:** Tom Sorahan, Linda Nichols

**Affiliations:** Institute of Applied Health Research, University of Birmingham, Edgbaston, Birmingham, UK; Department of Statistics, Mathematical Sciences Building, University of Warwick, Coventry, UK

**Keywords:** amyotrophic lateral sclerosis, motor neuron disease, magnetic fields

## Abstract

**Background:**

Many studies have investigated magnetic field exposure and the risks of motor neuron disease (MND). Meta-analyses have found positive associations but a causal relationship has not been established.

**Aims:**

To investigate the risks of MND and occupational exposure to magnetic fields in a large UK cohort.

**Methods:**

Mortality of 37 986 employees of the former Central Electricity Generating Board of England and Wales was investigated for the period 1987–2018. Employees were first employed in the period 1942–82 and were still in employment on the 1 November, 1987. Detailed calculations enabled estimates to be made of magnetic field exposures. Observed deaths were compared with expected numbers based on mortality rates for the general population of England and Wales and Poisson regression was used to calculate rate ratios (relative risks) for categories of lifetime, lagged (distant) and lugged (recent) magnetic field exposure.

**Results:**

Mortality from MND in the total cohort was similar to national rates (observed 69, expected 71.3, SMR 97, 95% CI 76–122). There were no statistically significant trends of risks increasing with lifetime, recent or distant magnetic field exposure, although positive associations were observed for some categories of recent exposure.

**Conclusions:**

The study did not find that the cohort had elevated risks of MND as a consequence of occupational lifetime exposure to magnetic fields, although a possible role for recent exposures could usefully be investigated in other datasets.

**Key learning points**

What is already known about this subject:

- Many epidemiological studies have investigated MND risks in relation to magnetic field exposure.
- Meta-analyses of these studies have shown positive associations.
- A causal relationship has not been established.

What this study adds:

- This large UK study has found no convincing evidence to support the hypothesis that occupational lifetime exposure to magnetic fields is a risk factor for MND.
- The study has not shown convincing evidence for a role of recent magnetic field exposures on MND risks but it does show some inconsistent positive findings for such an effect.
- Any role for recent exposures will need to be assessed in other studies.

What impact this may have on practice or policy:

- The new findings have not provided persuasive evidence that past working practises and current occupational exposure limits have increased the risk of MND.
- A pooled analysis of original findings from all available studies, enabling the harmonization of exposure assessments, exposure metrics, exposure categories and analytical techniques would assist interpretation.

## Introduction

Many studies have investigated exposure to extremely low-frequency electric and magnetic fields (ELF-EMF) and risks of motor neuron disease (MND), often referred to by its principal subtype, amyotrophic lateral sclerosis (ALS). Four overlapping meta-analyses are available. Two of these analyses [1,2] have been summarized previously [3]. More recently, Huss et al. included the findings of 20 studies, reporting a slightly increased risk for those exposed to higher levels of magnetic fields in comparison to those exposed to lower levels (RR 1.14, 95% CI 1.00–1.30) [4], and Jalilian et al. combined findings from 27 studies and found a weak statistically significant association between occupational exposure to ELF-MF and the risk of MND (RR 1.20, 95% CI 1.05–1.38) [5].

These meta-analyses give some support to the hypothesis that ELF-EMF exposure is a risk factor for MND, but convincing evidence requires dose–response effects from cohort studies based on quantitative exposure estimates. This research seeks new information on occupational magnetic field exposure and the risk of mortality from MND by examining data from an epidemiological study of electric utility workers in the UK. Earlier research on this cohort considered follow-up to the end of 2004 and 2010, respectively [3,6]; a further 8 years of follow-up data are now available.

## Methods

This analysis is based on the National Epidemiology File of the former Central Electricity Generating Board (CEGB) of England and Wales [7]. This pseudonymized file comprises work histories and death details for those male and female workers with a minimum of 6 months employment and some employment in the period 1973–82 (many employees were first hired in earlier decades). Earlier reports from this survey assumed that the first known job had been carried out since starting employment with the CEGB; the analysis reported here is based on a subset of this file, the 37 986 workers (34 926 males and 3060 females) who were still in employment on the 1 November 1987, and for whom full pre-1973 work histories were available from the industry’s later Computerised Personnel Information System (CPIS). Follow-up particulars were received from NHS Digital for the period 1987–2018; mortality data included underlying and multiple-cause (ICD) coding. At the end of this follow-up period, there were 11 642 deaths, 356 (1%) subjects had emigrated and 436 (1%) were untraced.

After removing duplicate work history records for the cohort under analysis, there were 294 565 unique records for employment in the period 1937–1993 showing employment dates and text entries for facility/plant/location and job titles. There were 96 112 work history records which related to jobs held prior to joining CEGB; no attempt was made to estimate exposures for these jobs because no reliable information is available on sources of magnetic fields at the relevant workplaces. Employment dates for CEGB work were checked for consistency. There were 943 records with a start date later than the end date, and for these records it was assumed that the start date was correct, and that employment was continuous. There were 4299 records where the end date of one job was later than the start date of the next job and for these records it was assumed that the start dates were correct and end dates were edited. There were 64 545 work entries for jobs at non-operational CEGB sites such as office buildings, training centres and research establishments; these jobs were not considered to have unusual levels of magnetic field exposure. Of the work histories at operational sites, there were 123 264 records relating to employment in one of 289 power stations and 8878 records relating to 96 substations or 92 transmission district offices. The remaining 1766 work records were either uninformative or could not be reconciled with the rest of the work history and these were left to one side. Job dictionaries, developed earlier by industry personnel, were available for 8799 job titles in power stations and 1722 job titles in substations and transmission district offices, and these dictionaries were used to categorize CPIS job titles into eleven categories of power station work [see Table 2, ref. 7] and four categories of work at the other operational sites (managers, fitters and clerical, engineers, and overhead linesmen).

The exposure protocol used to convert employment histories into exposure histories (power frequency 50 Hz fields) has been summarized before [6,7], and the original detailed account is also available [8].

Power stations were not fully specified for 14 825 work history entries (e.g. Aberthaw PS rather than Aberthaw A PS or Aberthaw B PS). For many of these entries the specific power station could be determined by reviewing the dates of operation of the relevant plants. For the remaining ambiguous entries, a composite exposure value derived from the relevant plants was adopted.

Individual employment histories were cross-referenced with the exposure assessments to obtain estimates of daily magnetic field exposure for the period 1952–1993. Cumulative occupational exposures together with exposure ‘windows’ (lagged and lugged exposures) were then calculated separately for each study subject.

Seven variables were considered in the risk modelling: attained age, sex, calendar year, estimated cumulative occupational exposure to magnetic fields, magnetic field exposures in the most recent ten years, magnetic field exposures received more 10 years ago, and socio-economic status (as judged by the ‘negotiating body’ that the employee was represented by in the 1970s and 1980s).

Individuals begin to contribute person-years-at-risk on the 1 November, 1987, and stop contributions on the earliest of date of death, date of embarkation, date last known alive, their 100 birthday or the 31 December, 2018. EPICURE software [9] was used (double precision DOS version 2.12 dated March, 2002) (i) to calculate standardized mortality ratios (SMRs) based on ‘external’ comparisons with mortality rates for England and Wales specified by gender, 5-year age-groups, and 5-year calendar periods, and (ii) to carry out Poisson regression modelling [10], providing estimates of relative risk for each category of magnetic field exposure compared with the baseline (lowest) category with and without adjustment for other variables. The approach adopted to calculate trend statistics has been described before [3].

The survey has ethics approval from a local ethics committee and the University of Birmingham has an active Data Sharing Agreement (DSA) with NHS Digital.

## Results

Table 1 shows observed and expected numbers of deaths and standardized mortality ratios (SMRs) for MND (underlying cause analysis) and All Causes by gender, socio-economic status and calendar period. Mortality from MND was close to expectation while there were significantly fewer deaths than expected from all causes. There was no relationship between MND mortality and socio-economic status, whereas for All Causes there was a marked gradient with an SMR of 45 in managers rising to 85 in industrial workers.

**Table 1.** Standardized mortality ratios (SMRs) by gender, socio-economic status and calendar period from motor neuron disease^a^ and All Causes in UK electricity supply industry workers with CPIS work history data (*n* = 37 986), 1987–2018

	Obs	Exp	SMR	(95% CI)
Motor neuron disease				
Males	>64	>66.0	99	(77–125)
Females	<5	<5.0	56	(93–184)
Managers	0	0.4	0	—
Engineers, scientists	23	23.1	100	(65–147)
Admin, clerical	8	6.0	133	(62–253)
Industrial workers^b^	38	41.7	91	(65–124)
1987–2000	13	15.2	86	(48–143)
2001–2010	22	27.6	80	(51–119)
2011–2018	34	28.5	119	(84–165)
Total	69	71.3	97	(76–122)
*All Causes*				
Males	11 027	14 969.5	74(***)	(72–75)
Females	613	770.7	80(***)	(73–86)
Managers	51	112.9	45(***)	(34–59)
Engineers, scientists	2573	4845.4	53(***)	(51–55)
Admin, clerical	980	1317.0	74(***)	(70–79)
Industrial workers^b^	8036	9464.9	85(***)	(83–87)
1987–2000	2560	3914.9	65(***)	(63–68)
2001–2010	3978	5353.1	74(***)	(72–77)
2011–2018	5102	6472.2	79(***)	(77–81)
Total	11 640	15 740.2	74(***)	(73–75)

***P < 0.001, () indicates deficit.

^a^Underlying cause of ICD-8 348, ICD-9 335.2 or ICD-10 G12.2.

^b^Including construction and maintenance workers.

Observed and expected numbers of deaths and standardized mortality ratios (SMRs) for MND are shown by estimated levels of lifetime, distant and recent occupational exposures to magnetic fields in Table 2. There were no significantly elevated SMRs and no statistically significant trends in SMRs across the three sets of findings.

**Table 2.** Standardized mortality ratios (SMRs) for motor neuron disease^a^ by estimated levels of lifetime, distant (lagged) and recent (lugged) cumulative magnetic field exposures from 37 986 workers in the ÚK electricity supply industry with CPIS work history data, 1987–2018

Exposure to magnetic fields (μT.y)^b^	Obs	Exp	SMR	95%CI
*Occupational cumulative (lifetime) exposure to magnetic fields*				
0–2.4	34	30.2	113	(79–156)
2.5–4.9	2	7.1	28(*)	(5–93)
5.0–9.9	13	11.2	116	(64–193)
10.0–19.9	9	12.3	80	(39–147)
≥20.0	11	10.5	105	(55–182)
*P*-value for trend^c^	0.73			
*Occupational cumulative exposure to magnetic fields received more than 10 years ago*				
0–2.4	34	31.2	109	(77–151)
2.5–4.9	3	7.4	40	(10–110)
5.0–9.9	12	11.4	106	(57–177)
10.0–19.9	9	11.9	76	(37–139)
≥20.0	11	9.5	116	(61–201)
*P*-value for trend^c^	0.86			
*Occupational cumulative exposure to magnetic fields received in most recent 10 years*				
Zero	54	58.6	92	(76–119)
0.01–0.4	7	4.0	174	(76–344)
0.5–1.9	4	3.3	123	(39–297)
≥2.0	4	5.4	74	(23–178)
*P*-value for trend^c^	0.88			

*P < 0.05, () indicates deficit.

^a^Underlying cause of death coded to ICD-9 348, ICD-9 335.2 or ICD-10 G12.2.

^b^One year refers to a working year, approximately 250 8-h shifts.

^c^Linearly weighted *P*-value.

The following internal analyses were able to introduce three additional cases of MND, where the disease was mentioned on the death certificate but not as the underlying cause of death. Rate ratios (RRs) for MND and for All Causes are shown in Table 3 for categories of estimated cumulative lifetime occupational exposure to magnetic fields. Findings in the left-hand columns were adjusted for age and gender (the partially adjusted model), and findings in the right-hand columns were additionally adjusted for calendar period and socio-economic status (the ‘fully’ adjusted model). For MND, most of the RRs are close to unity although a low RR (based on only two deaths) is shown for the second exposure category. For All Causes, there were significantly raised RRs for analyses that only adjusted for age and gender, but additional adjustment for calendar time and socioeconomic status lowered all the RRs and the final trend statistic was negative (RR of 0.98 per 10 uT.y). Further analyses (not shown in Table 3) indicated that most of the difference in the findings from the partially and fully adjusted models arose from the introduction of the socio-economic status variable and not from the introduction of the calendar time variable.

**Table 3.** Rate ratios for motor neuron disease^a^ and All Causes mortality by levels of estimated cumulative magnetic field exposure, from 37 986 workers in the UK electricity supply industry with CPIS work history data, 1987–2018

Exposure to magnetic fields (μT.y)^b^	Obs	RR^c^	(95 % CI)	RR^d^	(95% CI)
*Motor neuron disease*					
0.0–2.4	34	1.0		1.0	
2.5–4.9	2	0.24*	(0.06–0.99)	0.25	(0.06–1.04)
5.0–9.9	14	1.05	(0.56–1.96)	1.12	(0.58–2.15)
10.0–19.9	10	0.69	(0.34–1.40)	0.74	(0.36–1.56)
≥20.0	12	0.96	(0.49–1.85)	1.04	(0.51–2.11)
RR per 10 μT.y^e^		1.01	(0.86–1.18)	1.03	(0.88–1.21)
*All Causes*					
0.0–2.4	4 659	1.0		1.0	
2.5–4.9	1 135	1.09*	(1.02–1.16)	1.03	(0.96–1.10)
5.0–9.9	1 888	1.13***	(1.07–1.19)	1.01	(0.95–1.06)
10.0–19.9	2 115	1.13***	(1.07–1.19)	0.98	(0.93–1.04)
≥20.0	1 843	1.12***	(1.06–1.18)	0.94*	(0.89–1.00)
RR per 10 μT.y^e^		1.02***	(1.01–1.03)	0.98*	(0.97–1.00)

*P < 0.05, ***P < 0.001.

^a^Any part of death certificate coded to ICD-8 348, ICD-9 335.2 or ICD-10 G12.2.

^b^One year refers to a working year, approximately 250 8-h shifts.

^c^Analysed simultaneously with sex and attained age (<45, 45–49, 50–54, 55–59, 60–64, 65–69, 70–74, 75–79, 80–85 and 85–99 years).

^d^Analysed simultaneously with sex, attained age, calendar period (1987–1999, 2000–2009 and 2010–2018), and socioeconomic status as judged by negotiating body (managers+scientists+engineers, admin+clerical, industrial+construction workers).

^e^Five exposure categories scored by the mean value in each category, namely 0.57, 3.70, 7.49, 14.21 and 44.54 μT.y.

Corresponding findings for distant (lagged) and recent (lugged) exposures are shown in Table 4. These two exposure variables were always analysed simultaneously. As before the left-hand column adjusts for age and gender, and the right-hand column is additionally adjusted for calendar time and socio-economic status; the Table 4 shows the results of four separate analyses. For MND and distant exposures, there are no statistically significant findings and rate ratios are close to unity. For MND and recent exposures there were significantly elevated risks in the fully adjusted model for the first two exposed categories, but the overall trend statistic was negative (RR of 0.91 per 10uT.y). Further analyses (not shown in the Table 4) found the introduction of the calendar time variable had more influence on the different findings from the partially and fully adjusted models than did the introduction of socio-economic status. For All Causes and distant exposures, there were significantly raised RRs for analyses that only adjusted for age and gender, but additional adjustment for calendar time and socioeconomic status reduced the risks and the final trend statistic was negative (RR of 0.99 per 10 uT.y). There were highly significantly elevated RRs for All Causes mortality with recent exposures when the analysis only adjusted for age and gender, but RRs were reduced after additional adjustment for calendar time and socio-economic status.

**Table 4.** Rate ratios for motor neuron disease^a^ and All Causes mortality by estimated levels of distant (lagged) and recent (lugged) cumulative magnetic field exposures from 37 986 workers in the ÚK electricity supply industry with CPIS work history data, 1987–2018

Exposure to magnetic fields (μT.y)^b^	Obs	RR^c^	(95 % CI)	RR^d^	(95% CI)
*Motor neuron diseas*e					
*Occupational cumulative exposure to magnetic field received more than 10 years ago*					
0–2.4	34	1.0		1.0	
2.5–4.9	3	0.35	(0.11–1.16)	0.36	(0.11–1.17)
5.0–9.9	13	1.01	(0.52–1.93)	1.02	(0.52–2.00)
10.0–19.9	10	0.75	(0.37–1.55)	0.76	(0.36–1.61)
≥20.0	12	1.16	(0.58–2.28)	1.16	(0.56–2.38)
RR per 10 μT.y^e^		1.05	(0.90–1.23)	1.06	(0.90–1.25)
*Occupational cumulative exposure to magnetic fields received in most recent 10 years*					
Zero	56	1.0		1.0	
0.01–0.4	7	1.83	(0.81–4.17)	3.11*	(1.23–7.87)
0.5–1.9	5	1.66	(0.63–4.34)	3.08*	(1.03–9.18)
≥2.0	4	0.79	(0.27–2.37)	1.67	(0.46–6.02)
RR per 10 μT.y^f^		0.64	(0.19–2.16)	0.91	(0.23–3.60)
All Causes					
*Occupational cumulative exposure to magnetic fields received more than 10 years ago*					
0–2.4	4 839	1.0		1.0	
2.5–4.9	1 170	1.04	(0.98–1.11)	1.01	(0.94–1.08)
5.0–9.9	1 911	1.08**	(1.02–1.14)	1.00	(0.95–1.06)
10.0–19.9	2 029	1.06*	(1.00–1.11)	0.97	(0.92–1.03)
≥20.0	1 691	1.06*	(1.00–1.12)	0.95	(0.89–1.01)
RR per 10 μT.y^e^		1.01	(1.00–1.02)	0.99*	(0.97–1.00)
*Occupational cumulative exposure to magnetic fields received in most recent 10 years*					
Zero	9 532	1.0		1.0	
0.01–0.4	676	1.43***	(1.32 to1.55)	1.07	(0.98–1.17)
0.5–1.9	550	1.46***	(1.33–1.60)	1.07	(0.97–1.18)
≥2.0	882	1.43***	(1.32–1.54)	1.00	(0.91–1.10)
RR per 10 μT.y^f^		1.43***	(1.31–1.56)	0.97	(0.87–1.07)

*P < 0.05, **P < 0.01, ***P < 0.001.

^a^Any part of death certificate coded to ICD-9 348, ICD-9 335.2 or ICD-10 G12.2.

^b^One year refers to a working year, approximately 250 8-h shifts.

^c^Analysed simultaneously with sex and attained age (<45, 45–49, 50–54, 55–59, 60–64, 65–69, 70–74, 75–79, 80–85 and 85–99 years).

^d^Analysed simultaneously with sex, attained age, calendar period (1989–99, 2000–2009 and 2010–2018), and socioeconomic status as judged by negotiating body (managers+scientists+engineers, admin+clerical, industrial+construction workers).

^e^Five exposure categories scored by the mean value in each category, namely 0.57, 3.70, 7.43, 14.18 and 44.24 μT.y.

^f^Five exposure categories scored by the mean value in each category, namely zero, 0.18, 1.10 and 8.59 μT.y.

## Discussion

This cohort of UK electricity generation and transmission workers had no relationship between estimated occupational lifetime exposures to magnetic fields and the risks of MND. This was also the case for distant (lagged) exposures although there some positive findings for recent (lugged) exposures.

It was very unlikely that magnetic field exposure would have any discernible effect on overall All Causes mortality and the purpose of the Poisson regression analyses for All Causes mortality was to establish a model (partial adjustment or full adjustment) that would allow a fair test of the null hypothesis for MND and magnetic field exposure. The argument here assumes that a positive or negative trend between All Cause mortality and magnetic field exposure is more likely to be due to inadequate control of confounding variables or selection effects than magnetic field per se. The analyses shown in Tables 3 and 4 for All Cause mortality indicated that there was positive confounding from socio-economic status in the partially adjusted models but that adjustment for socio-economic status was enough to remove this confounding. The importance of adjusting for socio-economic status in this cohort has been noted previously [3,6]. Nevertheless, confident interpretation of the positive findings in the fully adjusted model for MND and recent exposures is difficult because there was no positive trend with level of recent exposures and the positive findings for two exposure categories in the internal analysis get very little support from the SMR analyses. It is possible that the positive findings in the fully adjusted Poisson regression analysis have arisen from the estimation of many regression coefficients from a relatively small number of exposed cases.

The study has a number of strengths including the large size of the cohort, long period of follow-up, full and detailed contemporaneous work histories, and detailed exposure assessments that were based on an understanding of the physics of magnetic field exposure. It was no longer necessary to assume that the job and place of work known to be followed in the early 1970s was also followed in earlier decades. The study also has several limitations. Patterns of work for each job (time spent in different parts of power stations) were assumed to be the same for different power stations, and this assumption will have produced errors. The study has other limitations; health outcomes are obtained from death certificate data and not hospital diagnoses, and the study lacks smoking and other lifestyle data.

This study aimed to limit the unwanted effects of multiple testing by having predetermined exposure categories and an analysis plan to give most importance to trend tests across exposure categories. The possible role of threshold effects (no effects at lower exposures) or saturation effects (same effects at moderate and higher exposures) were not considered.

The addition of this study to any future meta-analysis of the whole literature will probably bring the meta-RR closer to unity. It would seem reasonable, however, to give this study more importance than many other studies because it does not suffer from the types of bias that routinely afflict case-control studies (participation bias, recall bias etc) and the exposure assessment is more detailed than that available to many other cohort studies. It also seems likely that the job-exposure matrices used to estimate magnetic field exposure in many population-based studies have a very poor predictive value in estimating ‘true’ exposure, whereas a validation of power station exposures used in the current analysis and based on monitoring of 215 workers for a week at three power stations found a strong correlation (*r* = 0.86) between predicted and measured fields (see Table 9 of ref. 8). Additional meta-analyses are unlikely, however, to cast much further light on the topic of magnetic fields and MND. What would be much more useful would be a pooled analysis of original data, enabling the harmonization of exposure assessments, exposure metrics, exposure categories and analytical techniques. Such an initiative is being attempted, although the task of harmonizing exposure assessments is far from trivial [11].

In conclusion, the current UK study does not indicate that occupational lifetime magnetic field exposures are a risk factor for MND but the possible role of recent exposures would be worth investigating in the other available studies.
